# The developments and emerging trends of Autonomic Nervous System Research in Arrhythmia: a bibliometric study from 2004 to 2024

**DOI:** 10.3389/fnins.2025.1595253

**Published:** 2025-04-28

**Authors:** Tingting Chen, Yanfeng Yang, Kun Shi, Feifei Si, Yizhou Wen, Xiao Yang

**Affiliations:** ^1^Department of Pediatric Cardiology, Chengdu Women’s and Children’s Central Hospital, School of Medicine, University of Electronic Science and Technology of China, Chengdu, China; ^2^Department of Obstetrics, Chengdu Women’s and Children’s Central Hospital, School of Medicine, University of Electronic Science and Technology of China, Chengdu, China; ^3^Chengdu Integrated TCM and Western Medicine Hospital, Chengdu, China

**Keywords:** autonomic nervous system, arrhythmia, bibliometric, emerging trends, development

## Abstract

The role of the autonomic nervous system in cardiovascular diseases has increasingly attracted the attention of researchers. This study aims to review research on the autonomic nervous system in arrhythmias from 2004 to 2024, with a focus on understanding the development trends in this field. Data for this study were sourced from the Web of Science Core Collection. We constructed and analyzed bibliometric visualizations related to publication trends, countries/regions, institutions, journals, research categories, themes, references, and keywords. Over the past two decades, academic output related to the autonomic nervous system’s role in arrhythmias has grown, although global research distribution remains uneven. The United States leads in publication volume and is home to many high-output institutions, providing it with significant academic influence and fostering international collaboration. By summarizing high-citation literature, clustering keywords, and performing a “burst detection” analysis of keywords, we identified that the mechanisms and assessment methods for autonomic nervous system regulation are major research focuses. Recent hotspots include the psychopathology related to the autonomic nervous system and autonomic regulation therapies. As the biomedical field shifts toward precision medicine, future research trends are likely to focus on identifying precise biomarkers for assessing autonomic nervous system function and developing novel strategies to regulate it. These strategies may include correcting immune dysfunction, psychological interventions, and surgical treatments. This study suggests that ganglionated plexi ablation may represent the most transformative intervention strategy for the Autonomic Nervous System currently available, and highlights electrodermal activity as an evaluation index with considerable potential for widespread application.

## 1 Introduction

Arrhythmia is a significant global public health issue (Savelieva et al., 2023). The incidence of fetal arrhythmias is approximately 1–2% of all pregnancies (Veduta et al., 2021; Kharbanda et al., 2022). In the general population, atrial fibrillation (AF) affects 1–3% of individuals, with incidence rates rising to 9% among those over 65 years and up to 17% in individuals aged over 80 years (Murphy et al., 2017). The Global Burden of Disease report highlights that the prevalence of arrhythmias has steadily increased over the last three decades, based on assessments from 204 countries and regions worldwide (Roth et al., 2020).

The pathogenesis of arrhythmia is complex and multifactorial. Among various contributing factors, abnormal autonomic nervous system (ANS) activity plays a pivotal role in several cardiovascular diseases, including heart failure, arrhythmia, and ischemic heart disease (Florea and Cohn, 2014; Fukuda et al., 2015; Herring et al., 2019; Gopinathannair et al., 2024).

The ANS, comprising the sympathetic and parasympathetic nervous systems, is integral to regulating vital physiological functions, such as heart rate, respiration, digestion, blood pressure, and metabolism (Karim et al., 2023). These two branches work in balance to coordinate and control physiological activities (Olshansky et al., 2022; Salamon et al., 2023).

The heart receives dual innervation from both systems. Sympathetic nerve fibers, originating from the lateral horn neurons in the thoracic spinal cord (T1–T5), release norepinephrine, which binds to myocardial cell receptors, enhancing heart rate, contractility, and conduction speed, ultimately increasing cardiac output and blood pressure. Conversely, parasympathetic nerve fibers, originating from the cardiac vagus nerve in the medulla oblongata, release acetylcholine, which reduces heart rate, myocardial contractility, and conduction speed, thereby decreasing cardiac output and blood pressure (Herring et al., 2019; Shen and Zipes, 2014; Goldberger et al., 2019).

Under conditions such as coronary insufficiency and elevated endocardial pressure, the vagus nerve becomes vulnerable to ischemia, reducing its regulatory control over the ventricular myocardium. This imbalance heightens sympathetic nervous system excitability, facilitating arrhythmogenesis (Schomig, 1990). Elevated catecholamine secretion and enhanced excitability of β1-adrenergic receptors lead to increased activation of adenylate cyclase, thereby elevating intracellular calcium ion levels. The electrical current generated by sodium-calcium exchange mechanisms may contribute to ectopic activity, indicating a possible association between autonomic nervous system dysfunction and systolia ectopica. Hasdemir reports that high-frequency stimulation of the pulmonary artery during continuous dopamine infusion can induce ventricular premature beats and even ventricular tachycardia, which is characteristic of right ventricular outflow tract origin, in healthy volunteers (Hasdemir et al., 2009).

Cardiac autonomic nervous dysfunction exacerbates, leading to increased atrial wall tension and sympathetic nerve excitation. This results in enhanced atrial myoregulation, induction of atrial premature, and an increase in triggering activity. Additionally, parasympathetic nerve excitation predisposes the formation of reentrant circuits, thereby providing a substrate for the initiation of atrial fibrillation. Furthermore, catecholamine-induced inflammatory environments promote macrophages to release nerve growth factor, which regulates cardiac sympathetic nerve remodeling through β1 receptors on macrophages (Lyu et al., 2020). ANS remodeling in atrial fibrillation primarily involves excessive innervation and sprouting of both sympathetic and parasympathetic nerves.

Over the past two decades, research into the ANS’s role in cardiac regulation has grown, establishing neural circuits between the heart and central nervous system and identifying key targets for therapeutic interventions. These advancements have spurred the development of multidisciplinary neural regulation techniques, including electrical, magnetic, acoustic, optical, and thermal methods (Stavrakis et al., 2015; Markman et al., 2020; Xiang et al., 2023; Sato et al., 2020; Zhong, 2006; Sun et al., 2022). While some techniques have entered critical stages of clinical translation, many ANS modulation strategies remain in preclinical development or face challenges in progressing to clinical trials.

Although the role of the autonomic nervous system in the occurrence, maintenance, and treatment of arrhythmias has been extensively investigated, existing studies remain highly fragmented. Moreover, reviews in this domain frequently lack objective visual data support and are overly reliant on researchers’ subjective interpretations of the disciplinary framework. This reliance on researcher perspectives leads to heterogeneity and limits the ability to perform comprehensive analyses of the research landscape, identify hotspots, and delineate emerging frontiers. Therefore, a comprehensive examination and critical review of scientific literature in the field of autonomic function related to arrhythmic diseases is indispensable.

This study leverages bibliometric analysis to systematically evaluate publications on autonomic nervous function in arrhythmia over the past two decades (2004–2024). The primary objectives include:

1. To reveal research trends: By conducting a quantitative analysis of annual publications, core literature, key journals, and leading institutions, a systematic and comprehensive knowledge framework was established. This enhances the understanding of the current scientific foundation, clarifies the distribution of research outcomes, and identifies the developmental stage of this field, thereby providing a basis for future resource allocation.
2. To identify key hotspots: Through keyword co-occurrence analysis and the mapping of research hotspots over time, the current focal areas of research can be objectively determined, minimizing the subjective bias inherent in traditional reviews and offering potential new research directions for new scholars to the field.
3. To optimize scientific research collaboration: By analyzing the international cooperation network, the limitations of existing collaborations are exposed, facilitating the breaking down of disciplinary barriers and promoting cross-regional and interdisciplinary communication and cooperation.

## 2 Methodology and materials

### 2.1 Research method: bibliometrics

Bibliometrics, which emerged as an independent discipline in 1969 (Pritchard, 1969; Chen et al., 2024), is widely used to describe, evaluate, and predict research trends and hotspots in specific fields (Kaplan, 1973). This method focuses on the system and characteristics of published literature, employing techniques from mathematics, statistics, and other fields to conduct comprehensive analyses of the distribution structure, quantitative relationships, and evolving patterns within a particular domain. Additionally, bibliometric analysis uses various indicators to assess both the quality of the literature and the current state of research in that field (Chen C. et al., 2014; Pan et al., 2024). Bibliometrics offers a rigorous approach to analyzing large volumes of scientific information, utilizing quantitative data from citation databases on specific topics. These data include details about authors, keywords, publishing institutions, countries, and distribution trends. With the aid of modern computational tools, bibliometric analysis is often supplemented by graphical and visual representations, which enhance data interpretation and make the results more accessible and comprehensive.

Various software tools are available for bibliometric analysis, such as CiteSpace, VOSviewer, and R language. Among these, CiteSpace is an information visualization software developed by Dr. Chaomei Chen at Drexel University in the United States, using Java programming language (Chen, 2005). It supports multiple data formats, features an interactive interface, and includes various analysis algorithms. Using multidimensional, temporal, and dynamic citation analysis techniques, CiteSpace can create a variety of rich and visually appealing maps that provide abundant information and have been widely applied in the field of information science both domestically and internationally (Chen, 2017). VOSviewer, developed by Van Eck and Waltman (2009), builds and displays the structure, evolution, and relationships of a knowledge domain based on “network data” relationships. It is known for its simple mapping and strong graphical presentation capabilities. R language, an open-source software widely used in the statistical field, can not only analyze bibliometric data but also further process and analyze information extracted from CiteSpace and VOSviewer results to generate statistical charts according to research needs.

This study employed the bibliometric analysis tools CiteSpace (6.3.3, 32-bit), VOSviewer (6.19), and R (4.4.1) with the “bibliometrix” package,^1^ combined with in-depth literature analysis, to comprehensively examine and interpret the research theme.

### 2.2 Data retrieval

This study selected the Web of Science Core Collection (WOSCC) as the data source, specifically utilizing indexes from the Science Citation Index Expanded (SCIE) and the Social Science Citation Index (SSCI). The WOSCC dataset was chosen for bibliometric analysis due to its comprehensive nature, with citation reference information readily available for analysis (Chen, 2015). Furthermore, the dataset from WOSCC can be directly processed using mainstream bibliometric software such as CiteSpace and VOSviewer (Gaviria-Marin et al., 2018), eliminating the need for format conversion. This is essential for ensuring the integrity of knowledge mapping and analysis.

To enhance the comprehensiveness and accuracy of the data retrieval, a search strategy combining MeSH (Medical Subject Headings) terms and free keywords was employed (Table 1). Keyword merging incorporated synonyms, aliases, and both singular and plural forms to ensure high-quality results. The retrieval period spanned from January 1, 2004, to December 31, 2024. The document types selected for inclusion were Articles and Review Articles. The preliminary search yielded a total of 4,406 journal papers, including 3,407 original research articles and 999 review articles. The initial data screening process is outlined in Table 1.

**Table 1:** Summary of data source and selection.

Category	Specific standard requirements
Research database	Web of Science Core Collection
Citation indexes	SSCI, SCIE
Searching period	1st January 2004 to December 31th 2024
Language	English
Searching keywords	[TS = (“Arrhythmia*” OR “Arrythmia*” OR “Cardiac Arrhythmia*” OR “Cardiac Dysrhythmia*” OR “Sinus Arrhythmia*” OR “Sinoatrial Arrhythmia*” OR “Atrial Fibrillation*” OR “Auricular Fibrillation*” OR “Persistent Atrial Fibrillation*” OR “Familial Atrial Fibrillation*” OR “Paroxysmal Atrial Fibrillation*” OR “Atrial Flutter*” OR “Auricular Flutter*” OR “Bradycardia*” OR “Bradyarrhythmia*” OR “Extrasystole*” OR “Premature Beat*” OR “Premature Cardiac Complex*” OR “Premature Cardiac Complices” OR “Ectopic Heartbeat*” OR “Heart Block*” OR “Auriculo-Ventricular Dissociation*” OR “Auriculo Ventricular Dissociation*” OR “A-V Dissociation*” OR “A V Dissociation*” OR “Atrioventricular Dissociation*” OR “Long QT Syndrome” OR “Electrocardiogram QT Prolonged” OR “Parasystole” OR “Tachycardia*” OR “Tachyarrhythmia*” OR “Ventricular Fibrillation*” OR “Ventricular Flutter*”)] AND TS = (“Autonomic Nervous System*” OR “Vegetative Nervous System*” OR “Visceral Nervous System*” OR “Parasympathetic Nervous System*” OR “Sympathetic Nervous System*” OR “Vagus Nerve*” OR “Pneumogastric Nerve*” OR “Tenth Cranial Nerve*” OR “Cranial Nerve X” OR “Nerve X*” OR “Nervus Vagus”)
Document types	Articles, Review Articles
Data extraction	Export with full records and cited references in plain text format
Sample size	4,406 (Before manual screening)

### 2.3 Literature screening

The focus of this study is on the development of the autonomic nerve system in relation to arrhythmia. Therefore, the core research topic of all included literature must be closely aligned with both arrhythmia and the autonomic nervous system. To ensure that the literature included in the analysis was directly relevant to the study’s topic, a manual screening process was conducted following the initial literature retrieval to exclude articles that were not closely related. This process helped to determine the final set of articles for analysis.

**Figure 1:**
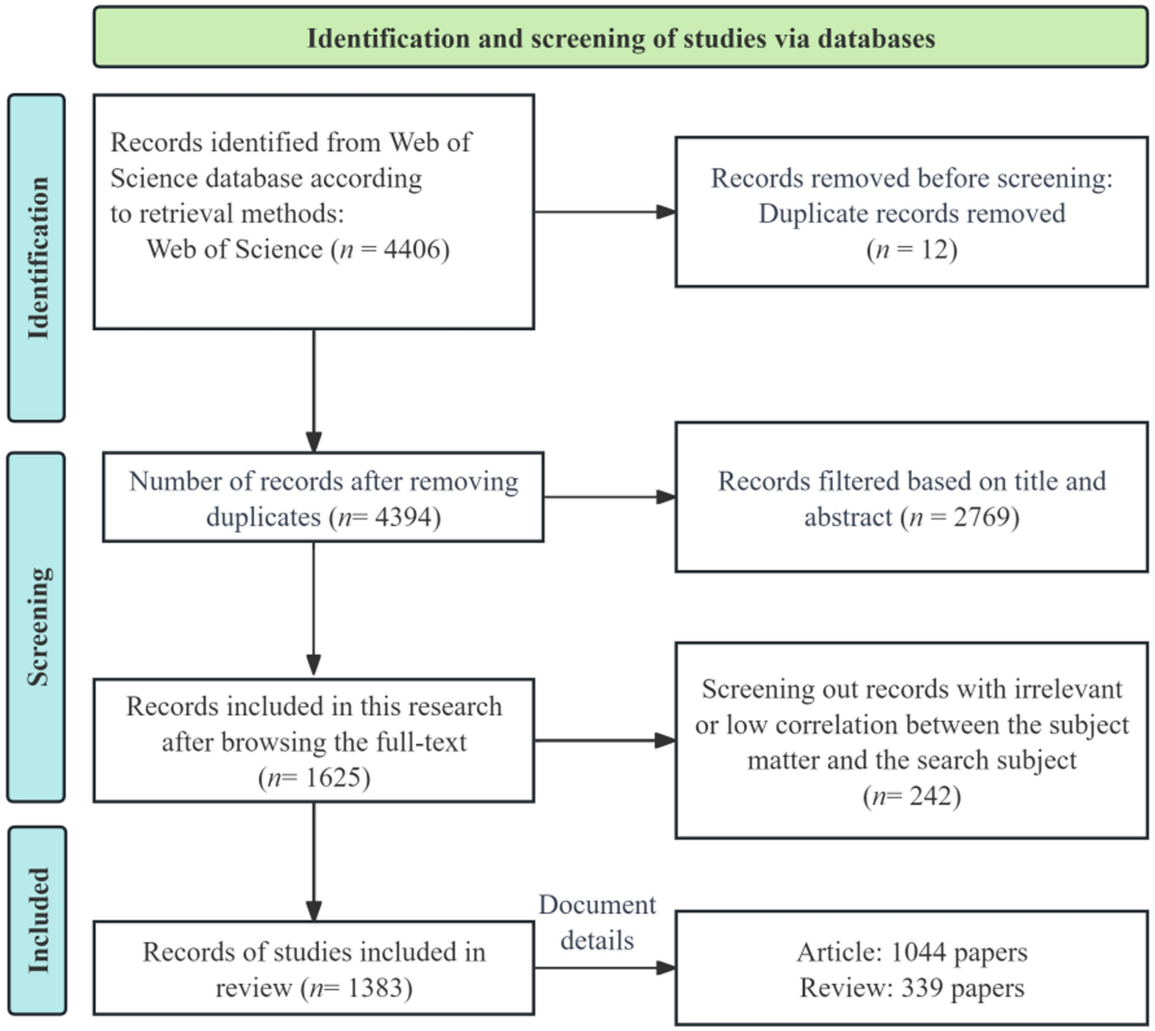
Flowchart for including studies to review.

The manual screening was performed in two stages. First, articles that were unrelated to the research topic were excluded based on their titles and abstracts. Following this initial screening, 1,625 articles were retained. This step was carried out by two members of the research team. Second, to ensure precise alignment with the research topic, three team members conducted a detailed full-text review. Based on the inclusion criteria outlined in Supplementary Table 1, 1,383 articles were ultimately selected for the final analysis, following the complete screening process as depicted in Figure 1.

### 2.4 Data standardization

In raw data exported from scientific databases, the same terms are often expressed in multiple ways. If these synonymous expressions are not addressed in bibliometric analysis, they can skew the results of the study (Nguyen and Hallinger, 2020). For instance, an author might be listed as both “Esler, Murray” and “Elser, Murray D.,” and keywords may appear as “pulmonary vein isolation” and “pulmonary veins isolation,” which should typically be merged. Therefore, before analyzing the screened literature, a data disambiguation process is necessary to standardize the data (Strotmann and Zhao, 2012; Van Eck and Waltman, 2019).

This study followed the data standardization process outlined in previous research (Taskin and Al, 2019) and performed the following steps:

1. The author and source fields were corrected and unified. If the same name appeared for different authors, a distinguishing identifier was added.
2. A check was conducted to identify whether any journals listed in the literature had undergone renaming in the past two decades, to avoid any potential impact of journal name changes on the analysis results.
3. The keyword field was standardized by unifying parts of speech and singular/plural forms, thereby reducing meaningless repetition in the knowledge map.

## 3 Performance analysis

### 3.1 Time trend of the publications

**Figure 2:**
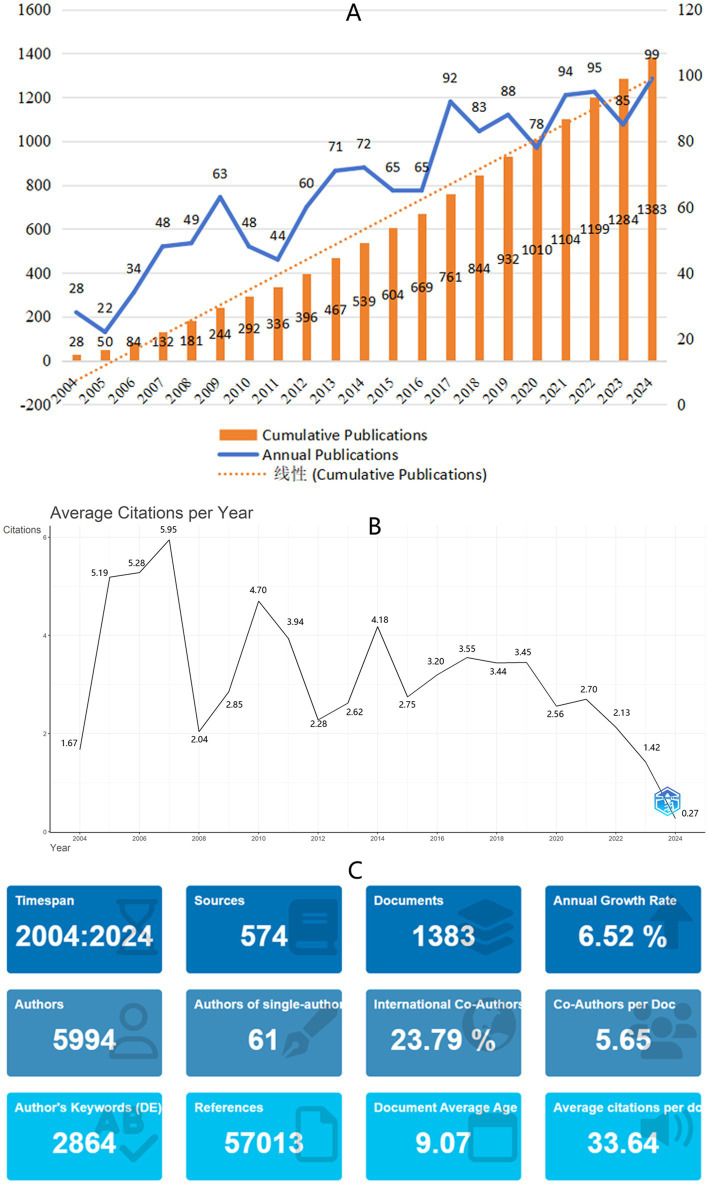
**(A)** Annual and cumulative global research publications of Autonomic Nervous System Research in Arrhythmia from 2000 to 2024. **(B)** Average citations per publication per year from 2000 to 2024. **(C)** Additional statistics on publications of Autonomic Nervous System Research in Arrhythmia from R bibliometrix.

From January 1, 2004, to December31, 2024, a total of 1,383 publications were retrieved from the WOSCC database (Figure 2A). The number of publications exhibited an overall upward trend, peaking in 2024 with 99 papers published. The annual growth rate of publications from 2004 to 2024 was 6.52%, and the cumulative number of publications followed a second-degree polynomial distribution (*y* = 68.779x - 159.48, *R*^2^ = 0.9826). The average citation rate per paper from 2004 to 2024 was 3.31, with the peak citation rate occurring in 2007 at 5.95 (Figure 2B). Figure 2C presents the references, journal count, and other statistical information for the publications included in this study.

### 3.2 Quantitative analysis of countries/regions

**Figure 3:**
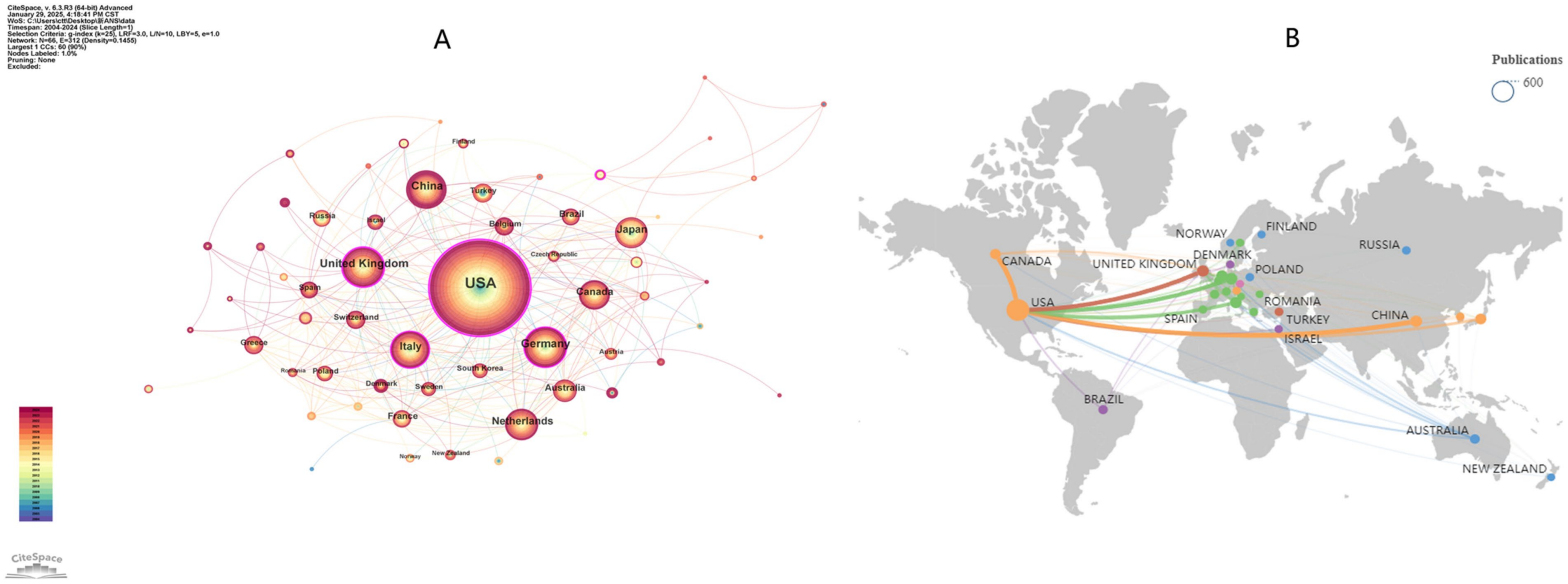
**(A)** Country/Region network visualization produced using, revealing links spanning across 66 countries/regions worldwide. **(B)** National/Regional collaboration network.

**Figure 4:**
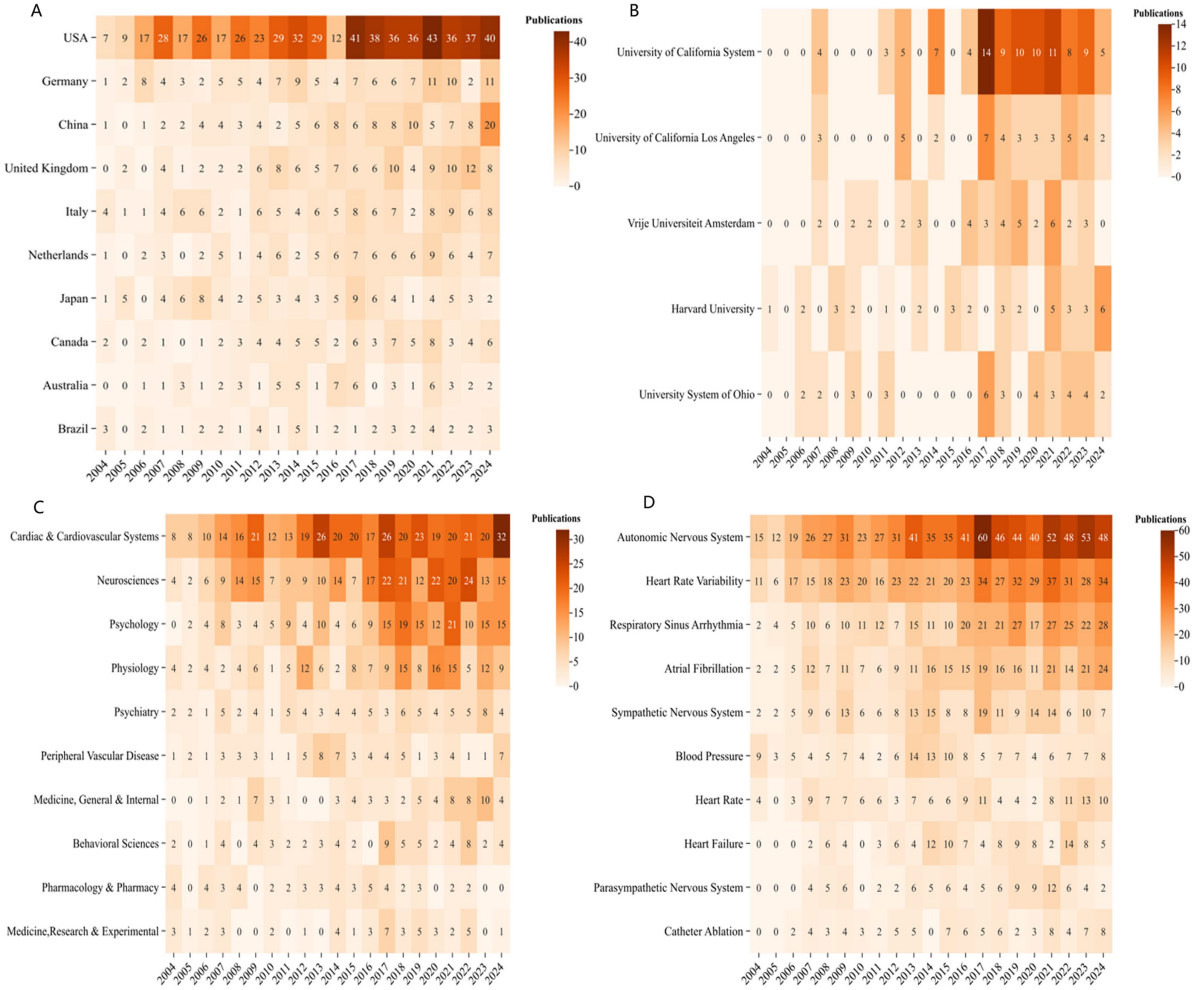
**(A)** Annual fluctuations in academic output for the high-productivity 884 countries/regions (top 10), **(B)** high-productivity institutions (top 5), **(C)** highfrequency 885 research categories (top 10), and **(D)** highfrequency keywords (top 10) from 2000 to 2024.

A total of 66 countries/regions contributed to the global scientific output in this study (Figure 3A). The top 10 countries/regions by publication output are listed in Table 2. Among them, the United States (*n* = 589), Germany (*n* = 119), and the China (*n* = 114) ranked first, second, and third, respectively. The United States (Betweenness Centrality, BC = 0.58), the United Kingdom (BC = 0.24), Italy (BC = 0.16), and Germany (BC = 0.15) are the four central nodes in the network (Figure 3A). Figure 3B illustrates the extensive collaborations between the United States and Europe, China, and Canada. Figure 4A depicts the annual publication trends for the top 10 countries, with the peak number of publications occurring in 2021 for most countries.

**Table 2:** Top 10 productive Countries/Regions of Autonomic Nervous System Research in Arrhythmia, ranked by the number of publications.

Ranking	Country/region	Count	Percentage	BC
1	United States	589	42.59%	0.58
2	Germany	119	8.60%	0.15
3	China	114	8.24%	0
4	United Kingdom	110	7.95%	0.24
5	Italy	105	7.59%	0.16
6	Netherlands	88	6.36%	0.07
7	Japan	85	6.15%	0.06
8	Canada	73	5.27%	0.05
9	Australia	53	3.83%	0.03
10	Brazil	44	3.18%	0.02

### 3.3 Quantitative analysis of institutions

**Figure 5:**
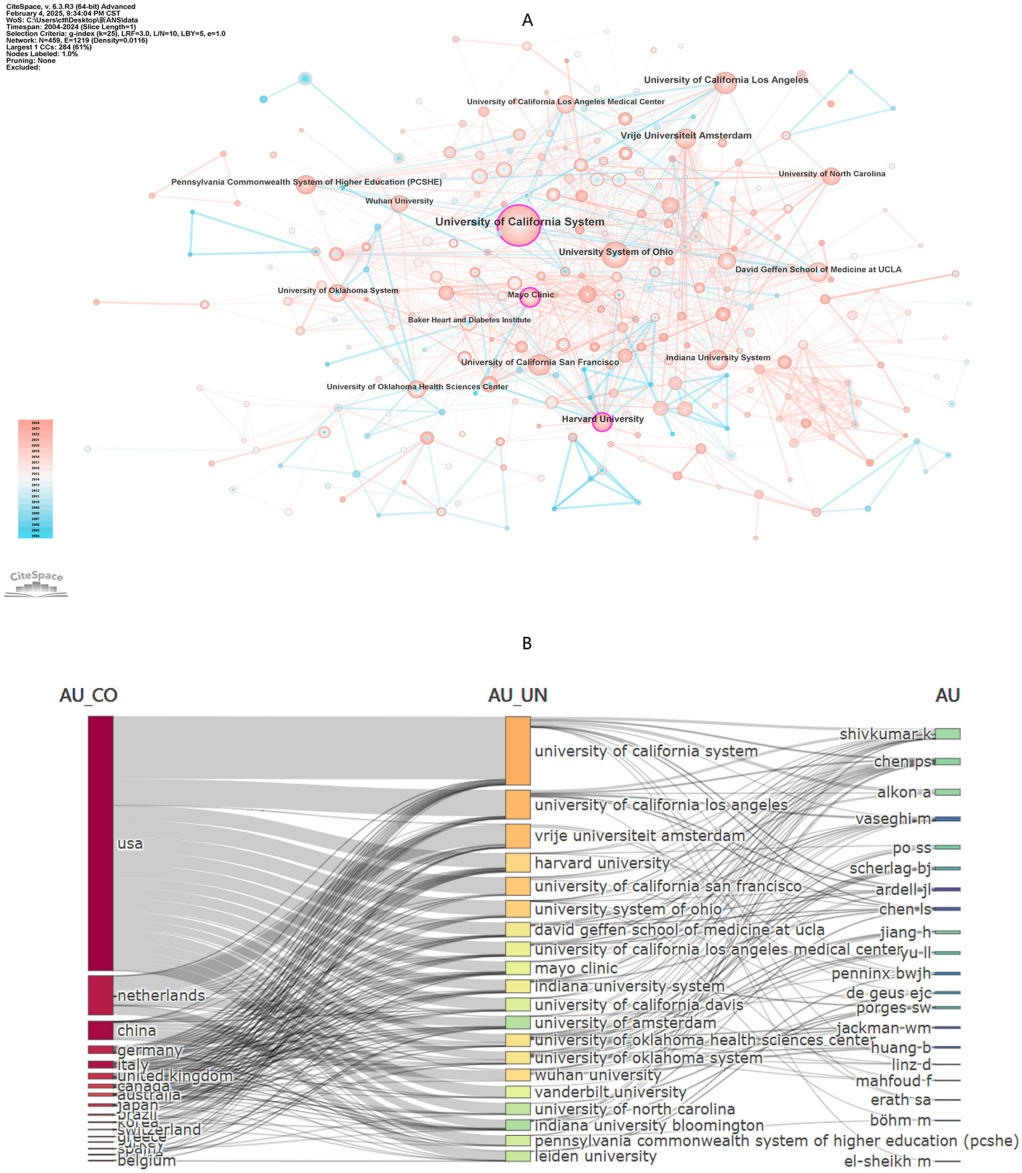
**(A)** Institution network visualization produced using CiteSpace, revealing 1,219 links spanning across 459 institutions worldwide. **(B)** Interconnections of the top 20 high productivity countries/regions, institutions and authors.

A total of 459 institutions contributed to the publications in this study. The top 10 institutions by number of publications are listed in Table 3. The University of California System leads with 99 papers, followed by University of California, Los Angeles with 41 papers, and theVrije Universiteit Amsterdam with 40 papers. Network visualization reveals three central institutions: Harvard University (Betweenness Centrality, BC = 0.12), the University of California System (BC = 0.12), and the Mayo Clinic (BC = 0.11) (Figure 5A). Figure 4B presents the annual publication trends of the top five institutions over time, with peak publication numbers occurring between 2017 and 2022. Figure 5B illustrates the cross collaborations among the top 20 countries, institutions and authors. It is evident that the most prominent research activity is concentrated in the United States and Europe.

**Table 3:** Top 10 productive institutions of Autonomic Nervous System Research in Arrhythmia, ranked by the number of publications.

Ranking	Institution	Count	Percentage	BC
1	University of California System	99	7.16%	0.12
2	University of California Los Angeles	41	2.96%	0.03
3	Vrije Universiteit Amsterdam	40	2.89%	0.07
4	Harvard University	38	2.75%	0.12
5	University System of Ohio	36	2.60%	0.07
6	University of California San Francisco	29	2.10%	0.02
7	Indiana University System	28	2.02%	0.06
8	David Geffen School of Medicine at UCLA	27	1.95%	0.02
9	Pennsylvania Commonwealth System of Higher Education (PCSHE)	25	1.81%	0.01
10	Mayo Clinic	24	1.74%	0.11

### 3.4 Quantitative analysis of journals

**Figure 6:**
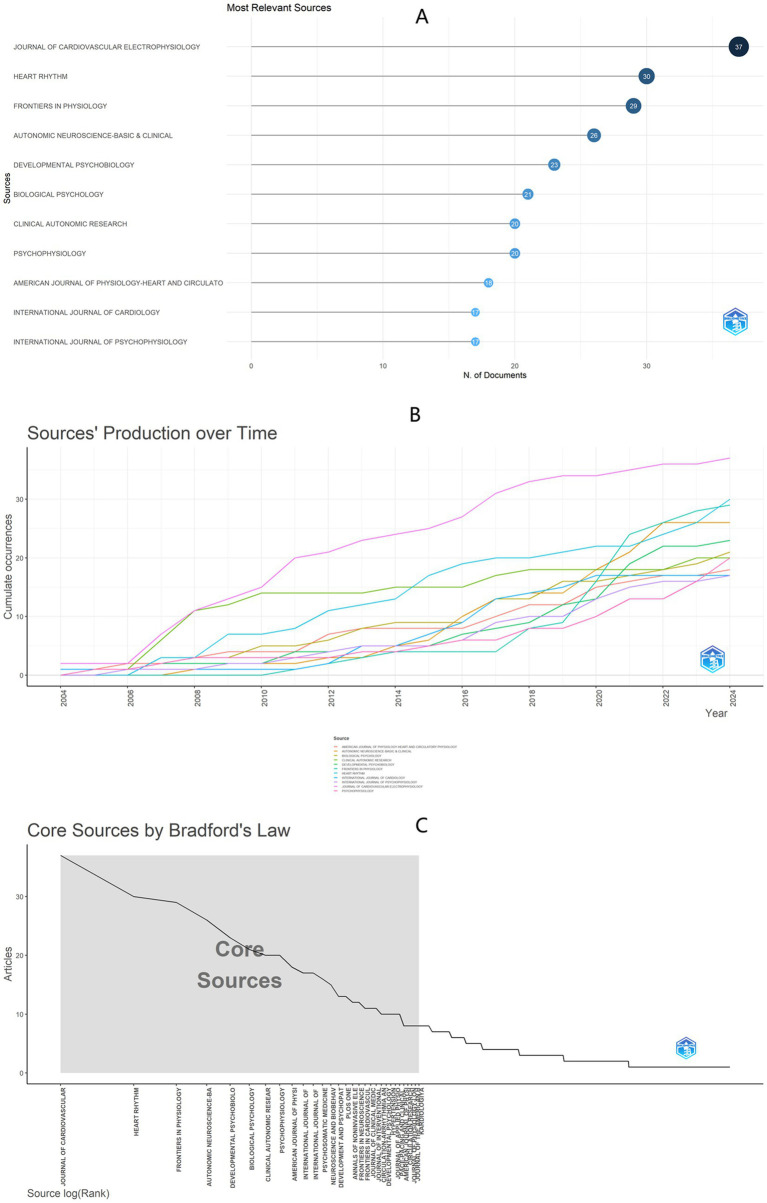
**(A)** Top 10 journals based on publication counts, including 2 journals sharing the 10th position. **(B)** Journal output trends within the top 10 from 2000 to 2024. **(C)** Delineation of core and non-core journals according to Bradford’s law.

A total of 574 journals contributed to global publications in this study (Figure 1C). Figure 6A presents the top 10 journals based on the number of publications, with most showing rapid growth since 2010 (Figure 6B). Among the top 10 journals, the Journal of Cardiovascular Electrophysiology leads with 37 publications, followed by Heart Rhythm (*n* = 30) and Frontiers in Physiology (*n* = 29). According to Bradford’s Law, the 574 journals are divided into three zones: Zone 1 consists of 30 core journals (Figure 6C; Table 4; Supplementary Table 2).

**Table 4:** Thirty core source journals in zone 1 according to Bradford’s law.

Ranking	Journal	Count
1	Journal of Cardiovascular Electrophysiology	37
2	Heart Rhythm	30
3	Frontiers in Physiology	29
4	Autonomic Neuroscience-Basic and Clinical	26
5	Developmental Psychobiology	23
6	Biological Psychology	21
7	Clinical Autonomic Research	20
8	Psychophysiology	20
9	American Journal of Physiology-Heart and Circulatory Physiology	18
10	International Journal of Cardiology	17
11	International Journal of Psychophysiology	17
12	Psychosomatic Medicine	16
13	Neuroscience and Biobehavioral Reviews	15
14	Development and Psychopathology	13
15	Plos One	13
16	Annals of Noninvasive Electrocardiology	12
17	Frontiers in Neuroscience	12
18	Frontiers in Cardiovascular Medicine	11
19	Journal of Clinical Medicine	11
20	Journal of Interventional Cardiac Electrophysiology	11
21	Circulation-Arrhythmia and Electrophysiology	10
22	Developmental Psychology	10
23	Hypertension	10
24	Journal of Applied Physiology	10
25	Pace-Pacing and Clinical Electrophysiology	10
26	American Journal of Physiology-Regulatory Integrative and Comparative Physiology	8
27	Circulation Research	8
28	Journal of Physiology-London	8
29	Journal of the American Heart Association	8
30	Kardiologiya	8

### 3.5 Quantitative analysis of subject categories

**Figure 7:**
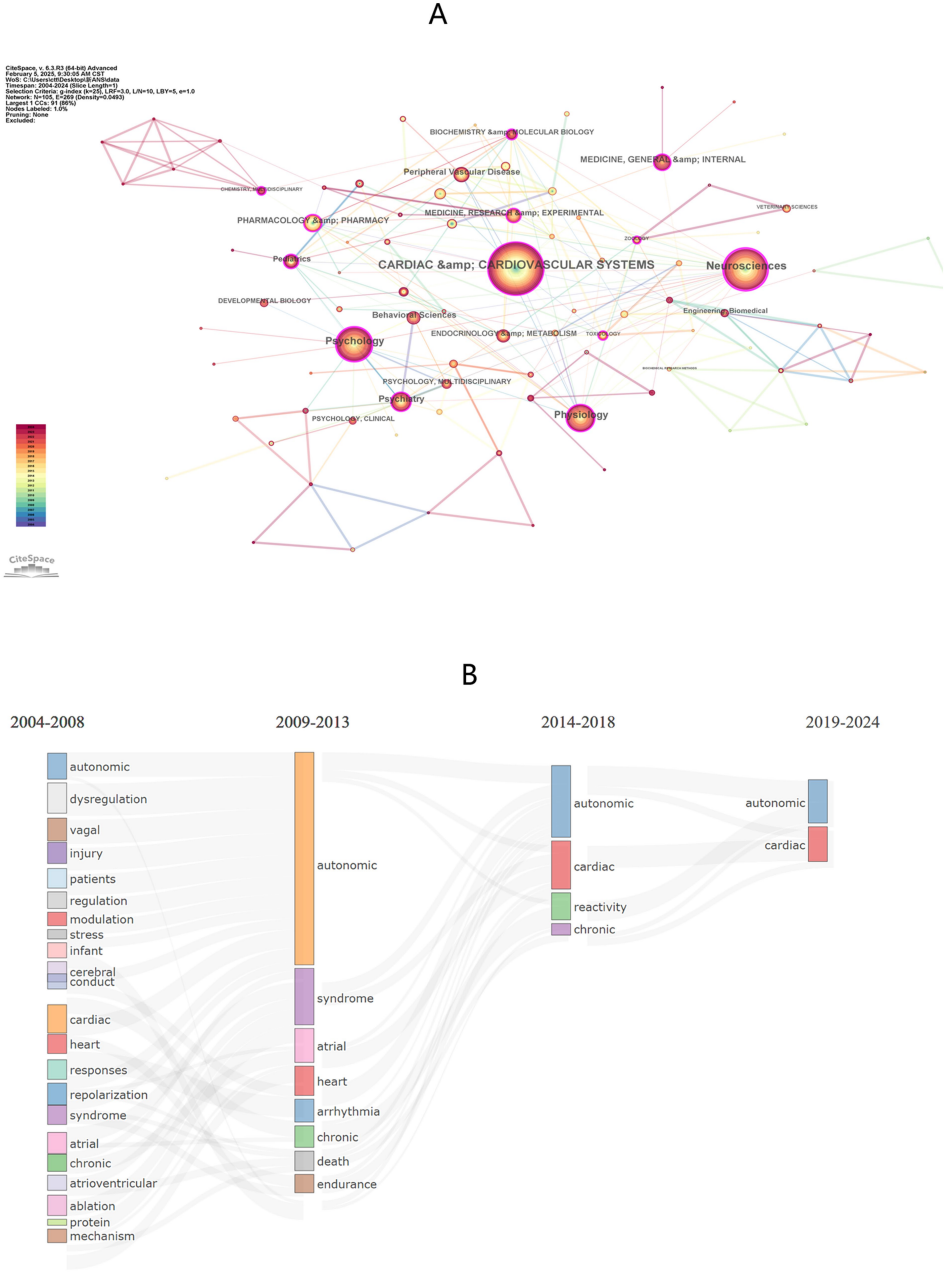
**(A)** The research category visualization produced using CiteSpace shows 269 links for all 105 research categories. Within the network visualization, 14 key nodes emerge as central: Neurosciences (BC = 0.54), Psychology (BC = 0.20), Physiology (BC = 0.18), Psychiatry (BC = 0.18), Medicine, Research and Experimental (BC = 0.17), Toxicology (BC = 0.15), Chemistry, Multidisciplianry (BC = 0.13), Cardiac and Cardiovascular Systems (BC = 0.12), Pharmacology and Pharmacy (BC = 0.11), Pediatrics (BC = 0.11), Biochemistry and Molecular biology (BC = 0.11), Zoology (BC = 0.11). **(B)** R bibliometrics-thematic evolution tool traces the progression of research themes in the realm of Autonomic Nervous System Research in Arrhythmia, spanning from 2004 to 2024. The time segmentation points were established in 2008, 2013, and 2018.

CiteSpace identified 105 research categories (Figure 7A). The top three categories with the highest publication counts are Cardiac & Cardiovascular Systems (*n* = 395), Neuroscience (*n* = 272), and Psychology (*n* = 190) (Table 5). The network visualization reveals 12 central nodes, with the top four being Neuroscience (BC = 0.54), Psychology (BC = 0.20), Psychiatry (BC = 0.18), and Physiology (BC = 0.18). Figure 4C illustrates the annual publication trends for the top 10 categories, showing that peak output for most categories occurred in 2021–2024.

**Table 5:** Top 10 productive subject categories of Autonomic Nervous System Research in Arrhythmia, ranked by the number of publications.

Ranking	Subject category	Count	BC	Year
1	Cardiac and Cardiovascular Systems	395	0.12	2004
2	Neurosciences	272	0.54	2004
3	Psychology	190	0.20	2005
4	Physiology	152	0.18	2004
5	Psychiatry	82	0.18	2004
6	Medicine, General and Internal	68	0.10	2006
7	Peripheral Vascular Disease	68	0.04	2004
8	Behavioral Sciences	66	0	2004
9	Pharmacology and Pharmacy	50	0.11	2004
10	Medicine, Research and Experimental	46	0.17	2004

### 3.6 Quantitative analysis of research themes

Figure 7B illustrates the progression of research themes over the past 20 years, divided into 5-year intervals. From 2004 to 2008, there were 22 distinct research themes. Between 2009 and 2013, the number of themes narrowed to 8, including topics such as autonomic nervous system, heart rate variability, syndrome, chronic orthostatic assessment, and atrial fibrillation stimulation. During this period, new themes emerged, including respiratory sinusarrhythmia, sudden death risk, and endurance personality athletes, replacing some of the earlier themes.

**Figure 8:**
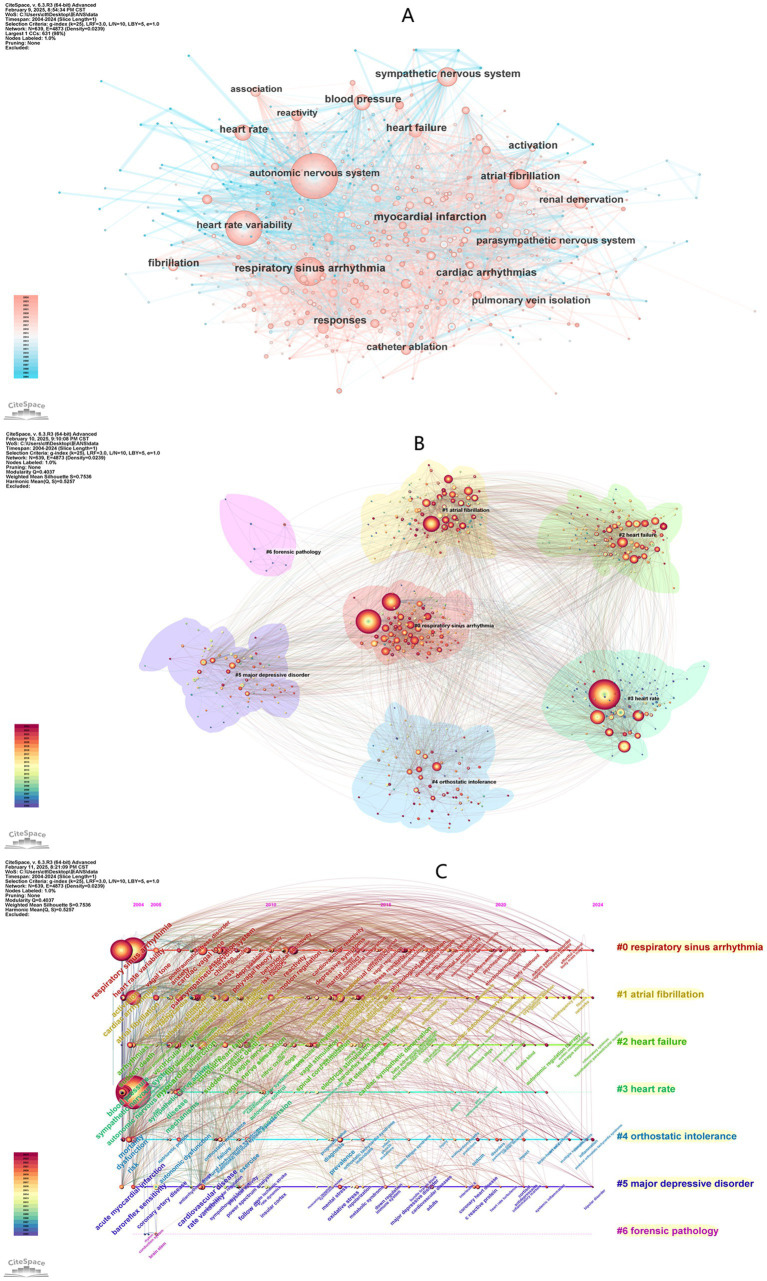
**(A)** Keyword network visualization produced using CiteSpace, revealing 4,873 links spanning across 639 keywords. **(B)** Cluster information for keywords reveals the plausibility and reasonability of the cluster structure, as indicated by the Q score and S score. **(C)** Keyword timeline visualization map for Autonomic Nervous System Research in Arrhythmia.

From 2014 to 2018, these themes continued to evolve, with a primary focus on the autonomic nervous system. Additionally, research on parasympathetic reactivity to stress expanded rapidly. In the most recent period, 2019–2024, only two prominent themes remained. The autonomic nervous system continues to be a significant and widely explored area, while cardiac atrial fibrillation appears to be a area that may be in the process of declining research interest. The keyword timeline visualization map (Figure 8C) clearly illustrates this trend.

It is important to note that the R bibliometric theme evolution tool may not fully capture the intrinsic connections between themes within the same period, or these connections may be relatively weak. However, this does not negate the existence of internal relationships. Supplementary Figures 1–4 offer additional clarification for Figure 7B.

### 3.7 Quantitative analysis of references

**Figure 9:**
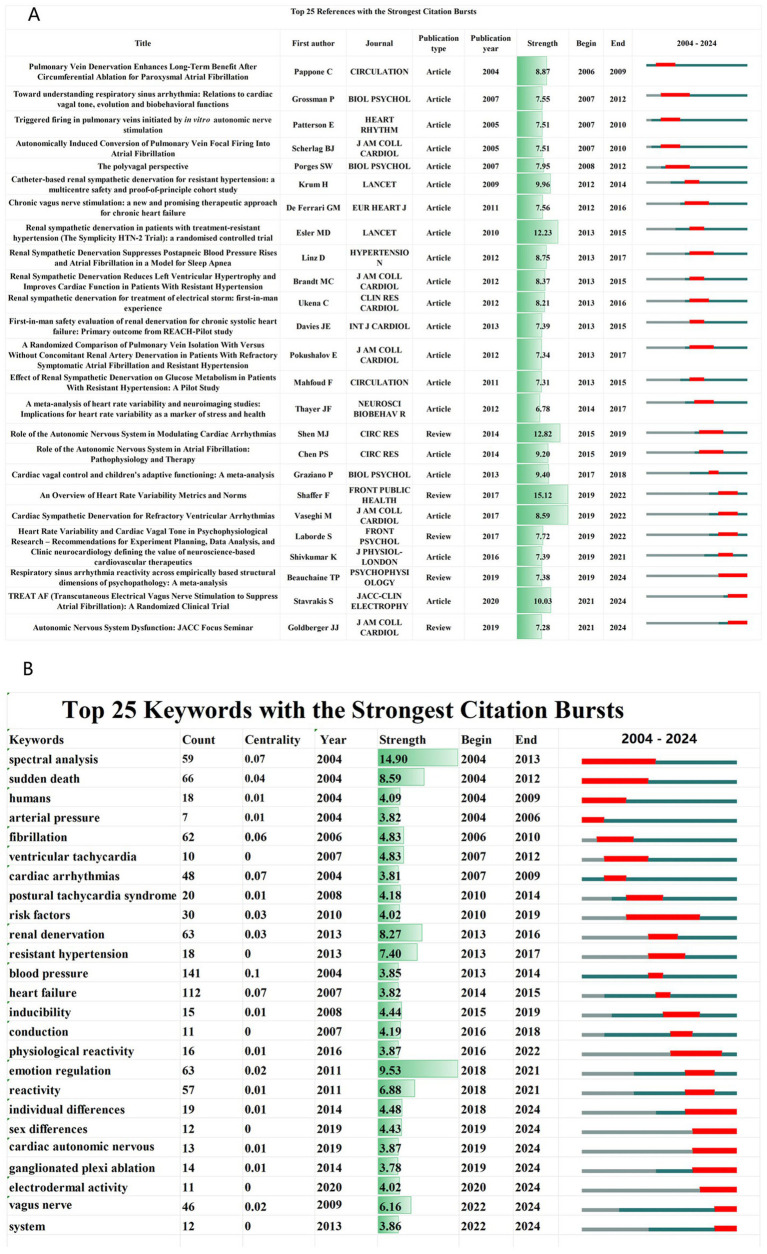
**(A)** Top 25 references with the strongest citation bursts, including information on first authors, journals, and publication types, arranged in ascending order based on burst time. **(B)** Top 25 keywords with the strongest citation bursts, including information on count, centrality (considered here as BC), and year, arranged in ascending order based on burst time.

This study involved 57,013 references (Figure 1C). References with high burst strength often have a greater impact on subsequent research and scientific output. The top three references in terms of burst strength are Heart Rate Variability (burst strength = 15.12) (Shaffer and Ginsberg, 2017), Autonomic Nervous System (burst strength = 12.82) (Shen and Zipes, 2014), and Renal Sympathetic Denervation (burst strength = 12.23) (Esler et al., 2010) (Figure 9A).

### 3.8 Quantitative analysis of keywords

A total of 639 keywords were extracted, with no central nodes identified in the current network (Figure 8A). The top 10 most frequently occurring keywords are listed in Table 6, with their research activity peaking between 2021 and 2024 (Figure 4D). The three most frequent keywords are Autonomic Nervous System (*n* = 744), Heart Rate Variability (*n* = 488), and Respiratory Sinus Arrhythmia (*n* = 311).

**Table 6:** The top 10 keywords with the highest frequency in of Autonomic Nervous System Research in Arrhythmia.

Ranking	Keyword	Frequency	BC	Year
1	Autonomic nervous system	744	0.05	2004
2	Heart rate variability	488	0.06	2004
3	Respiratory sinus arrhythmia	311	0.09	2004
4	Atrial fibrillation	260	0.07	2004
5	Sympathetic nervous system	191	0.08	2004
6	Blood pressure	141	0.10	2004
7	Heart rate	136	0.08	2004
8	Heart failure	112	0.07	2007
9	Parasympathetic nervous system	93	0.05	2007
10	Catheter ablation	84	0.04	2006

These 639 keywords are grouped into 7 clusters (Figure 8B; Supplementary Table 3), with the clustering results demonstrating strong reliability and credibility (modularity score, *Q* = 0.4037; silhouette score, *S* = 0.7536). The two most active clusters are Cluster 0 (Respiratory Sinus Arrhythmia) and Cluster 1 (Atrial Fibrillation), each containing more than 100 keywords. The keyword timeline visualization highlights the evolution of research trends over time (Figure 8C).

Initially, the research focus centered on Atrial Fibrillation and Heart Rate Variability. However, it has since shifted to emerging fields such as Psychopathology, Autonomic Regulation Therapy, and areas related to Psychosocial Development in Childhood. By detecting sharp bursts in keyword frequency over short periods, this study identifies emerging research hotspots and frontiers (Wang et al., 2024). Figure 9B presents the top 25 keywords ranked by burst strength.

## 4 Discussion and implications

### 4.1 Analysis of the geographical distribution of publications

#### 4.1.1 Global academic landscape in autonomic nervous system research

The top 10 countries/regions and institutions contributing to research on autonomic nervous system (ANS) function predominantly belong to the Americas, Europe, and Asia. With the exception of China and Brazil, these countries are all developed nations, emphasizing the correlation between economic power and academic output. Developed countries and high-level institutions possess significant advantages and influence in this field, with the United States leading in academic contributions and Europe following closely. This dominance highlights the substantial academic influence of these regions.

This geographical distribution aligns with observations from organizations such as the American Autonomic Society, the American Academy of Neurology, and the International Federation of Clinical Neurophysiology, which have jointly published guidelines on the electrodiagnostic assessment of the ANS (Cheshire et al., 2021). To advance research and integrate findings into clinical practice, these organizations, along with the International Society for Autonomic Neuroscience and the European Federation of Autonomic Societies, have facilitated international collaboration and reached consensus on definitions of autonomic disorders and methods for ANS testing (Cheshire et al., 2021; Zhao and Jin, 2017; Gibbons et al., 2014; Freeman et al., 2011).

#### 4.1.2 Historical milestones and notable contributions

The United States has maintained a leading role in ANS research, excelling in both academic achievements and clinical applications. For example, Figure 3A illustrates early U.S. contributions, such as the 1949 study conducted at the Presbyterian Hospital of New York, titled “Autonomic Effects on Stimulating Rostral Portion Cingulate Gyri in Man” (Pool and Ransohoff, 1949).

In 2004, cardiologists from the San Raffaele Hospital in Milan, Italy, reported that pulmonary vein denervation enhances the long-term benefits of circumferential ablation for paroxysmal atrial fibrillation. This finding linked reduced vagal tone with the success of circumferential pulmonary vein ablation (CPVA) in preventing arrhythmias (Pappone et al., 2004).

Further advancements came from Roy Freeman at Harvard Medical School in 2006, who reviewed the assessment of cardiovascular autonomic function. Freeman highlighted the ANS’s critical role in arrhythmias, sudden cardiac death, sleep disorders, hypertension, obesity, and other conditions. His work clarified the clinical implications of ANS dysfunction and proposed directions for its clinical assessment (Freeman, 2006).

Psychological research has also significantly contributed to this field, with studies addressing mental disorders, cognitive impairments (Agelink et al., 2002; Pham et al., 2021), and parenting (Alen et al., 2022).

#### 4.1.3 Impact of the COVID-19 pandemic

The COVID-19 pandemic has emerged as a significant factor influencing ANS research. Infection with COVID-19 exacerbates autonomic dysfunction in many patients (Pan et al., 2021). This period also marked a surge in publications, with 2021 recording the highest number. Most of the top 10 countries/regions in terms of academic output experienced a significant increase in publications during this time, underscoring the pandemic’s impact on the field.

#### 4.1.4 Challenges and opportunities for underdeveloped regions

Global academic output reveals a pronounced regional distribution. Factors such as inadequate research funding, weak infrastructure, limited collaboration, and lack of confidence hinder the academic competitiveness of underdeveloped countries/regions. Without disruptive events, the academic influence of developed nations remains robust.

However, globalization and healthcare expansion offer promising opportunities. Cross-national and cross-institutional collaborations between developed and underdeveloped regions are expected to increase, fostering knowledge transfer and innovation. These collaborations can help underdeveloped regions overcome existing challenges, enhancing their contributions to the field of autonomic nervous system research.

### 4.2 Overview of knowledge maps and research hotspots

The evolution of research themes in autonomic nervous function among arrhythmia patients reflects a dynamic and progressive process. To better analyze these changes, the timeline has been divided into four periods:

- January 2004–December 2008
- January 2009–December 2013
- January 2014–December 2018
- January 2019–July 2024

The keyword networks for each period were analyzed to understand the shifting focus of research and the maturing understanding of the field.

#### 4.2.1 2004–2008: fragmented beginnings

During this initial phase, the volume of research was relatively low. Key areas of focus included:

- The pathophysiology of heart failure (Fox et al., 2007; Olshansky et al., 2008).
- Cardiac autonomic dysfunction caused by diabetes (Boulton et al., 2005; Maser and Lenhard, 2005).
- The effects of emotional and psychological factors on cardiac autonomic modulation (Carney et al., 2005; Meerlo et al., 2008; Bensafi et al., 2003).

Research was largely fragmented, with topics ranging from descriptive studies to preliminary investigations of mechanisms. Keywords such as sympathetic nervous system (Kario, 2004), atrial fibrillation (Lőrincz et al., 2008), and aggression (Dodge, 2006), etc., reflecting a nascent understanding of the field.

#### 4.2.2 2009–2013: focused exploration

This period marked a reduction in the breadth of research themes, with a stronger focus on specific topics, including:

- Arrhythmia and respiratory sinus arrhythmia (Numata et al., 2013).
- The regulatory role of the autonomic nervous system during endurance exercise (Fürholz et al., 2012).

New keywords emerged, such as sudden cardiac death (Billman, 2009), cortisol (Schumacher et al., 2013), and ion channels (Volders, 2010), indicating a deeper dive into specialized areas. This trend reflects a shift from broad overviews to focused investigations.

#### 4.2.3 2014–2018: critical exploration phase

The number of annual publications during this phase suggests that research on autonomic nervous function in arrhythmias entered a critical exploration stage. Key developments included:

- Refinement of disease-specific insights: Research became more detailed, exploring autonomic changes in various disease states. Studies highlighted topics such as reducing inflammatory responses through autonomic balance (Koopman et al., 2017) and the harmful effects of tobacco smoke exposure on the autonomic nervous system (Middlekauff et al., 2014).
- Mechanisms of autonomic activation in arrhythmias: Studies delved into the role of autonomic activation in triggering arrhythmias and its potential anti-arrhythmic effects. Researchers identified specific autonomic triggers and their impact on cardiac electrophysiology (Shen and Zipes, 2014; Chen P. et al., 2014).
- Adolescents and mental health: The scope of research expanded to include the autonomic nervous function of adolescents, particularly its relationship with depressive symptoms, anxiety (Dieleman et al., 2014), suicide (Koenig et al., 2015), and internet addiction (Kim et al., 2016). This phase also explored underlying factors (Hu et al., 2016) and potential interventions (Yaroslavsky et al., 2016).

#### 4.2.4 2019–2024: maturation and refinement

The volume of publications continued to grow, and research themes matured further. Notable influences and developments during this period included:

- Impact of the COVID-19 pandemic (2020–2022): The pandemic accelerated research in autonomic dysfunction. Studies demonstrated that enhanced sympathetic nerve activity during SARS-CoV-2 infection, combined with hypoxemia, increased cardiac workload. This mismatch in oxygen supply and demand led to arrhythmogenic substrate release (Del Rio et al., 2020). Additionally, autonomic dysfunction emerged as a potential contributor to post-COVID-19 syndrome symptoms such as fatigue, palpitations, and cognitive impairment (Dani et al., 2020; Dotan et al., 2022).
- Specialization of research topics: Research themes became more specific. For instance, the keyword autonomic nervous system diseases evolved into more detailed terms like postural orthostatic tachycardia syndrome (Mueller et al., 2023). New elements such as psychophysiology and cardioneuroablation (Pachon-M et al., 2020) also gained prominence.

These trends reflect a deeper and more nuanced understanding of autonomic nervous function, signaling the maturation of the field.

### 4.3 Analysis of future trends and implications

#### 4.3.1 Advancements in understanding autonomic nervous function and arrhythmias: a 20-year perspective

Over the past two decades, research on autonomic nervous function in arrhythmia patients has centered on the mechanisms and assessment of autonomic nervous imbalance. A major development has been the widespread use of Heart Rate Variability (HRV) as a non-invasive method to evaluate autonomic nervous system (ANS) activity. HRV analyzes the variability between successive heartbeats in sinus rhythm, reflecting the regulatory effects of the ANS on cardiac rhythm and its correlation with physiological states. Notably, reduced HRV has been established as a predictor of increased all-cause cardiovascular and/or arrhythmic mortality (Sztajzel, 2004). The evolution of research topics depicted in Figure 7B indicates a declining trend in HRV research, thereby highlighting the necessity to develop more reliable indicators for assessing autonomic function.

Heart rate turbulence (HRT) (Schmidt et al., 1999), which reflects the rapid autonomic nervous system modulation following ventricular premature beats, has been confirmed to predict the mortality rate of myocardial infarction (Bauer et al., 2005). In contrast, HRT observed after atrial premature beats (Lindgren et al., 2003) differs from that seen after ventricular premature beats, potentially being more influenced by the vagus nerve.

The integrated physiological theory of respiratory sinus arrhythmia highlights respiration as a primary factor influencing cardiovascular variability, including heart rate, blood pressure, and sympathetic nervous tension. Normal respiratory cycles involve minor accelerations and decelerations of heart rate, regulated by the combined actions of the sympathetic and parasympathetic nervous systems (Grossman et al., 2004).

#### 4.3.2 ANS, inflammation, and gut microbiota

Inflammatory responses, immune dysregulation, and gut microbiota alterations significantly affect ANS function. Dysbiosis in gut microbiota and the accumulation of harmful metabolites are linked to excessive sympathetic activation, increased norepinephrine release, platelet aggregation, macrophage recruitment, and foam cell formation, leading to atherosclerosis, myocardial ischemia, and ventricular fibrillation (Allison and Ditor, 2014; Bellocchi et al., 2022; Hinterdobler et al., 2021).

Recent studies suggest that gut microbes such as Enterococci and *Escherichia coli* produce H2S, a gaseous neurotransmitter that influences the sympathetic nervous system, cardiovascular system, and immune system (Ayaz and Zubcevic, 2020). Modulating gut microbiota and its metabolites offers a promising approach to managing ANS dysfunction and related cardiovascular diseases. However, the complexity of the gut microbiome necessitates further studies to optimize beneficial microbiota manipulation while minimizing adverse food-microbiota interactions.

#### 4.3.3 Emotional regulation and wearable technologies

Research has increasingly explored the intersection of emotional regulation and ANS activity, revealing that prolonged activation of the “defense-alert” response, linked to cognitive fixation (e.g., worry and rumination), is a significant contributor to autonomic imbalance (Ottaviani et al., 2015). HRV serves as an indicator of the activity and balance of ANS, as well as its capacity to respond to internal and external stimuli. This metric can effectively reflect an individual’s emotional regulation ability.

In addition to HRV, Figure 9B illustrates electrodermal activity (EDA), a rapidly expanding field of research. EDA involves recording autonomic nervous system functional activity via surface-based signals of autonomic nervous activity, providing an objective measure of cognitive-emotional arousal and the autonomic regulation of sweat glands in the skin (Shah et al., 2021; Grasser et al., 2022). The non-invasive and convenient nature of EDA detection offers a robust theoretical foundation for the development of wearable devices aimed at monitoring emotions, mental health, and autonomic nervous activity, showcasing broad application potential (Yu et al., 2019; Dark et al., 2022).

#### 4.3.4 Innovative therapeutic approaches

New perspectives in the treatment of arrhythmias involve modulation of the ANS. Vagus nerve stimulation (VNS), approved by the FDA for refractory depression, has shown efficacy in reducing ventricular arrhythmias and improving cardiac function post-myocardial infarction (Chen et al., 2023). Similarly, Low-Intensity Focused Ultrasound (LIFU) targeting the dorsal anterior cingulate cortex (dACC) has been effective in alleviating pain perception and modulating cardiac autonomic responses (Strohman et al., 2024).

Low-Intensity Ultrasound Modulation applied to cardiac sympathetic ganglia has shown promise in inhibiting sympathetic activity and reducing ventricular arrhythmias (Wang et al., 2020).

Low-level transcutaneous vagus nerve stimulation (T-VNS) has been demonstrated to elicit both antiadrenergic and anticholinergic effects (Sha et al., 2011), thereby prolonging the effective atrial refractory period. Furthermore, it attenuates the activity of the cardiac autonomic nervous system, interrupting the pathological cycle induced by excessive activation of this system and associated atrial remodeling (Yu et al., 2011). As a result, T-VNS serves as an effective intervention for inhibiting the onset of atrial fibrillation (Stavrakis et al., 2015).

The ganglionated plexi (GP), located within the epicardial fat pad, serve as a critical interface between the endogenous and exogenous components of the cardiac autonomic nervous system. Ablation of the GP reduces variability in the effective atrial refractory period and diminishes the incidence of pulmonary vein-induced atrial fibrillation (Lemola et al., 2008). It not only enhances the safety and efficacy of atrial fibrillation ablation procedures (Pokushalov et al., 2010), but also provides substantial clinical benefits for treating atrial fibrillation in patients with concurrent bradycardia (Hada et al., 2018). Performing a “burst detection” analysis of keywords, Figure 9B indicates that research on ganglionated plexi ablation has experienced rapid growth over the past 5 years. This suggests that GP ablation holds significant clinical application potential as a novel treatment for ANS.

Stellate ganglion blockade (SGB) provides a reversible block of the sympathetic nerves innervating the heart, thereby modulating autonomic nervous dysfunction induced by heightened sympathetic activity and ameliorating the imbalance between sympathetic and vagal tone. This intervention produces an effect analogous to that of beta-blockers (Fudim et al., 2016) and may serve as a therapeutic option for arrhythmias, including ventricular tachycardia (Cardona-Guarache et al., 2017).

#### 4.3.5 Electroimmunology: bridging electrophysiology and immunology

Damage to the cardiac ANS alters immune cell distribution, phenotype, and function. Neurons release neurotransmitters, neuropeptides, and cytokines that modulate immune cells, while immune imbalances affect ANS morphology and functionality. Immune cells also influence myocardial electrophysiology through ion channels and gap junctions, participating in arrhythmogenic processes.

The emerging field of electroimmunology bridges electrophysiology and immunology, providing a framework for understanding immune mechanisms in arrhythmias (Grune et al., 2021). Targeting neuroimmune crosstalk represents a promising strategy for arrhythmia treatment, underscoring the potential for therapies that integrate insights from both domains.

## 5 Conclusions, limitations, and prospects

### 5.1 Conclusions and future research

#### 5.1.1 Global trends and future directions in research on autonomic nervous function in arrhythmias (2004–2024)

This study employed bibliometric methods to systematically collect and analyze global research on autonomic nervous function in arrhythmias from 2004 to 2024. By visualizing trends in publication volume, research activity by countries and institutions, prominent journals, influential references, and keyword networks, the study offers a comprehensive overview of the field. This approach is designed to assist scholars in tracing the historical development of autonomic nervous system research, identifying critical literature, and clarifying emerging research hotspots. It aims to guide researchers in identifying future directions and opportunities within this domain.

#### 5.1.2 Key findings and implications

##### 5.1.2.1 Role of the autonomic nervous system in arrhythmias

As research has advanced, it has become increasingly evident that autonomic nervous system imbalance plays a pivotal role in the pathogenesis of arrhythmias. Through visualization analysis, the study revealed that the mechanisms underlying arrhythmias involve diverse biological functions, target genes, and signaling pathways associated with the autonomic nervous system. This highlights its critical influence on disease progression and suggests that the autonomic nervous system could serve as:

- A biomarker for diagnosing arrhythmias.
- An indicator for assessing treatment efficacy and prognosis.
- A potential clinical intervention target for arrhythmia management.

##### 5.1.2.2 Challenges and opportunities in clinical application

Despite the potential of the autonomic nervous system as a therapeutic target, significant challenges remain. These include:

A. Understanding mechanisms: The influence of the autonomic nervous system on arrhythmias involves a multitude of mechanisms. For instance, neurotransmitters such as norepinephrine, released into the myocardium during sympathetic nerve excitation, may enhance the automaticity of Purkinje fibers and trigger excitability by activating L-type Ca2 + channels in the inhibitory domain of the Purkinje fiber K + channel, thereby inducing ventricular arrhythmias (Ziegelstein, 2007; Pinto, 1999). Additionally, alterations in sympathetic nerve excitability may serve as one of the triggering factors for atrial fibrillation (Li et al., 2017), though its underlying mechanisms remain to be elucidated. The characteristics of vagal nerve remodeling following myocardial infarction also warrant further investigation.

Consequently, researchers need to deepen their understanding of how the autonomic nervous system functions and contributes to arrhythmogenesis.

B. Developing accurate evaluation indicators: Currently, HRV, the most widely used non-invasive indicator for evaluating autonomic nervous function, is susceptible to interference and influence from respiratory activity, motor functions, and other autonomic nervous system-related diseases, thereby limiting its clinical application to some extent (Tiwari et al., 2021; Schwerdtfeger et al., 2019). Given the distinct responses of heart rate behavior to atrial premature beats and ventricular premature beats, the calculation formula of HRT, a widely utilized indicator in prognostic assessments for patients with heart disease, requires corresponding adjustments (Lindgren et al., 2003).

Creating reliable and precise indicators for assessing autonomic nervous function is essential to advance diagnostic and therapeutic approaches.

C. Designing effective interventions: Autonomic nerve stimulation has been widely used in the treatment of cardiovascular diseases. Notably, low-level transcutaneous auricular vagus nerve stimulation (LL-TaVNS) shows promising potential for treating cardiovascular and inflammatory conditions. However, it may cause bradycardia in some cases. To confirm its reliability and safety for clinical use, further research and large-scale trials are needed (Zou et al., 2024).

The newly developed hybrid nanogenerator can adjust stimulation parameters in real-time according to the individual’s physiological state, automatically deliver stimulation pulses during atrial fibrillation onset, and establish a closed-loop, self-powered vagus nerve modulation system to optimize therapeutic efficacy while minimizing side effects (Sun et al., 2022).

Based on the thermal effect of ultrasound, transcatheter ultrasound ablation for atrial fibrillation treatment (Lins et al., 2010) and renal sympathetic nerve ablation for hypertension management (Azizi et al., 2018; Fengler et al., 2019) have been implemented with promising outcomes. However, long-term efficacy varies significantly among individuals. Further research is needed to determine the optimal ultrasound ablation target for future clinical applications.

There is a pressing need to design strategies that regulate autonomic nervous function with minimal side effects, ensuring safety and efficacy.

##### 5.1.2.3 Emerging strategies for arrhythmia management

Modulation of the autonomic nervous system has emerged as a promising therapeutic strategy. Successful outcomes in clinical trials—spanning pharmacological interventions, targeted therapies, and surgical approaches—indicate significant progress in this area. Key directions for future development include:

- Drug development: Medications that effectively modulate autonomic nervous system activity.
- Interventional techniques: Innovative, minimally invasive methods for autonomic nervous system regulation.
- Surgical methods: Advanced techniques such as neuromodulation surgeries tailored to patient-specific needs.

#### 5.1.3 Conclusion

From 2004 to 2024, the global research landscape of arrhythmic autonomic function has undergone significant transformation, offering valuable insights into its pathogenesis and potential therapeutic applications. Key aspects include:

- The mechanisms and evaluation methods of ANS regulation have been central to research efforts. Recent focal areas encompass psychopathological associations with the ANS and autoregulatory therapeutic approaches.
- Future research directions are likely to emphasize the identification of precise biomarkers for assessing ANS function and the development of novel modulation strategies, such as immune dysfunction correction, psychological interventions, and surgical treatments. This study highlights that GP ablation represents the most promising ANS intervention strategy, while EDA serves as a widely potential utilized indicator for ANS assessment.
- Despite existing challenges, breakthroughs in the assessment, treatment, and regulation of arrhythmias via the ANS will be realized through strengthened basic-clinical collaboration, enhanced cross-regional and interdisciplinary communication, as well as integrated research efforts.

### 5.2 Limitations

This study has certain limitations that must be considered. The analysis was based exclusively on the core collection of the Web of Science databases. Consequently, significant documents from other databases, such as SCOPUS and EBSCO, were not included, potentially limiting the comprehensiveness of the results. To provide a more complete understanding of global research trends, future studies should incorporate a broader range of databases.

Given the extensive number of keywords involved in autonomic nerve research, a few keywords might be overlooked due to variations in terminology, incomplete coverage of specialized terms, and other factors. Therefore, we will focus on continuously refining our retrieval strategy in the future.

In conclusion, despite its limitations, this study offers valuable and comprehensive information for researchers and institutions. It provides a foundation for understanding the developmental trends of research on autonomic nervous function in arrhythmia and serves as a reference point for future studies aimed at advancing knowledge and clinical applications in this field.

## Data Availability

The original contributions presented in the study are included in the article/Supplementary material, further inquiries can be directed to the corresponding author.

## Footnotes

^1^ https://www.bibliometrix.org
