# Supplementary figures and images for: The developments and emerging trends of Autonomic Nervous System Research in Arrhythmia: a bibliometric study from 2004 to 2024
Source: Front Neurosci. 2025 Apr 28;19:1595253. doi: 10.3389/fnins.2025.1595253 (PMC12066699; doi:10.3389/fnins.2025.1595253)

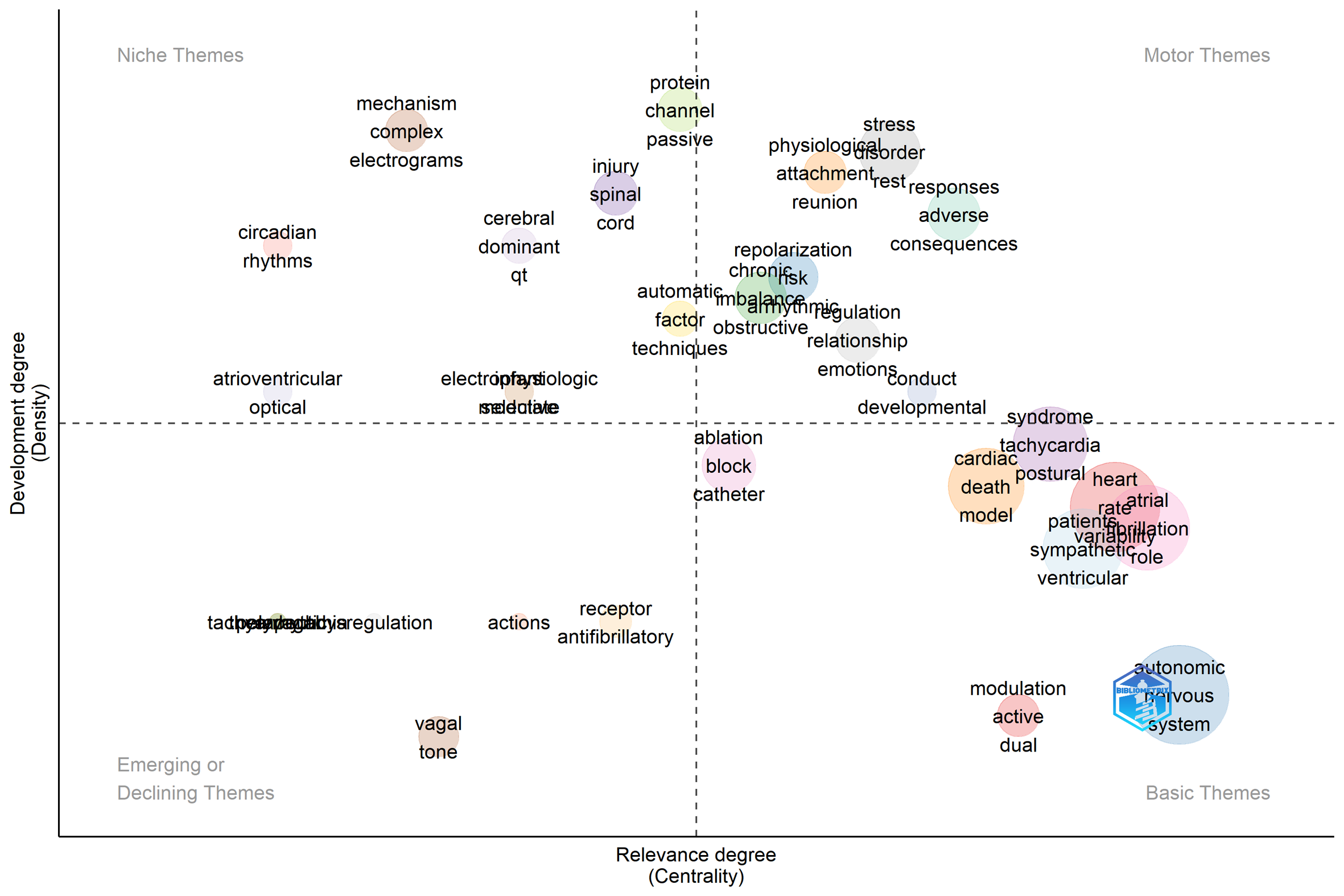

Supplement: Supplementary file 1 [file Image_1.TIF]

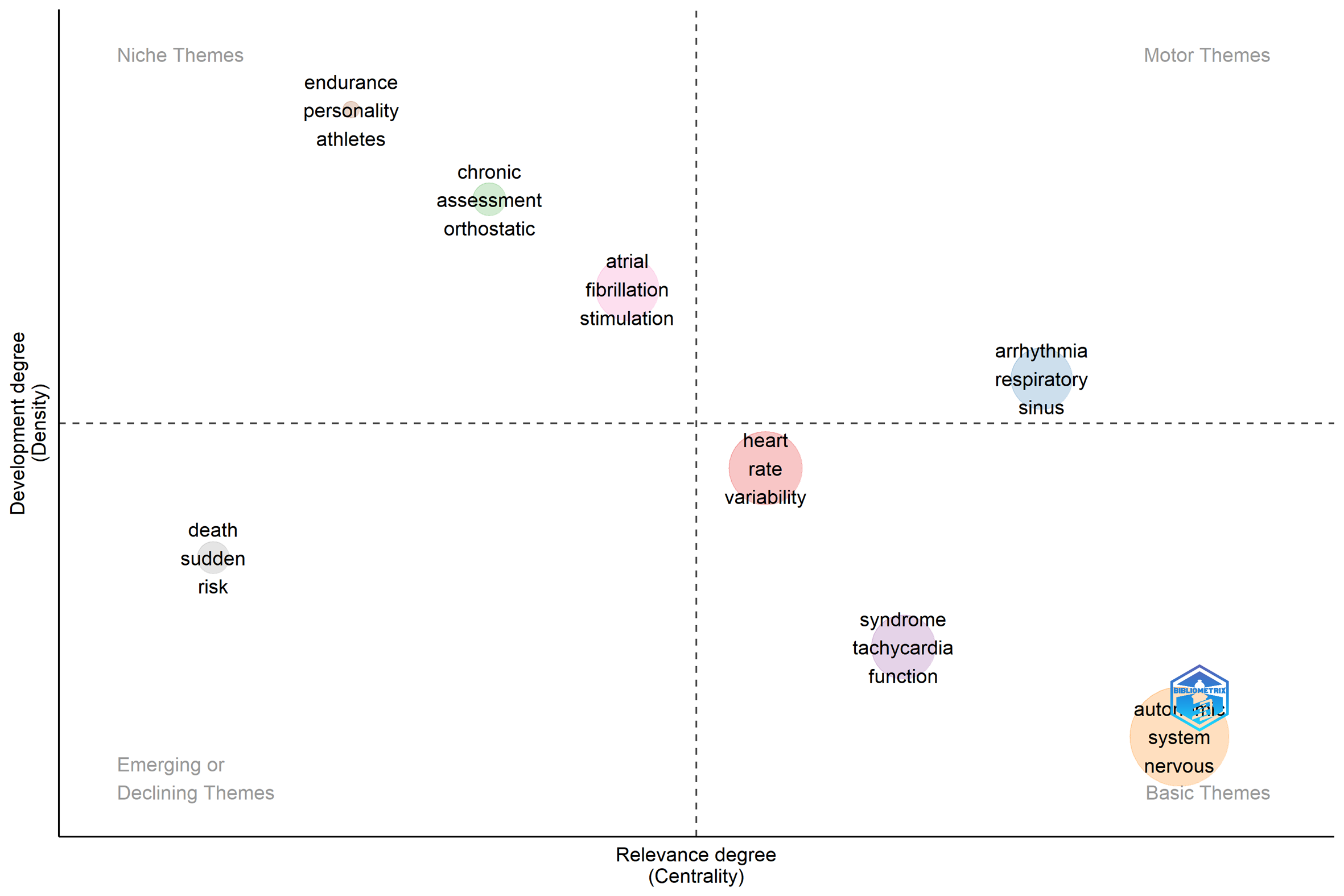

Supplement: Supplementary file 2 [file Image_2.TIF]

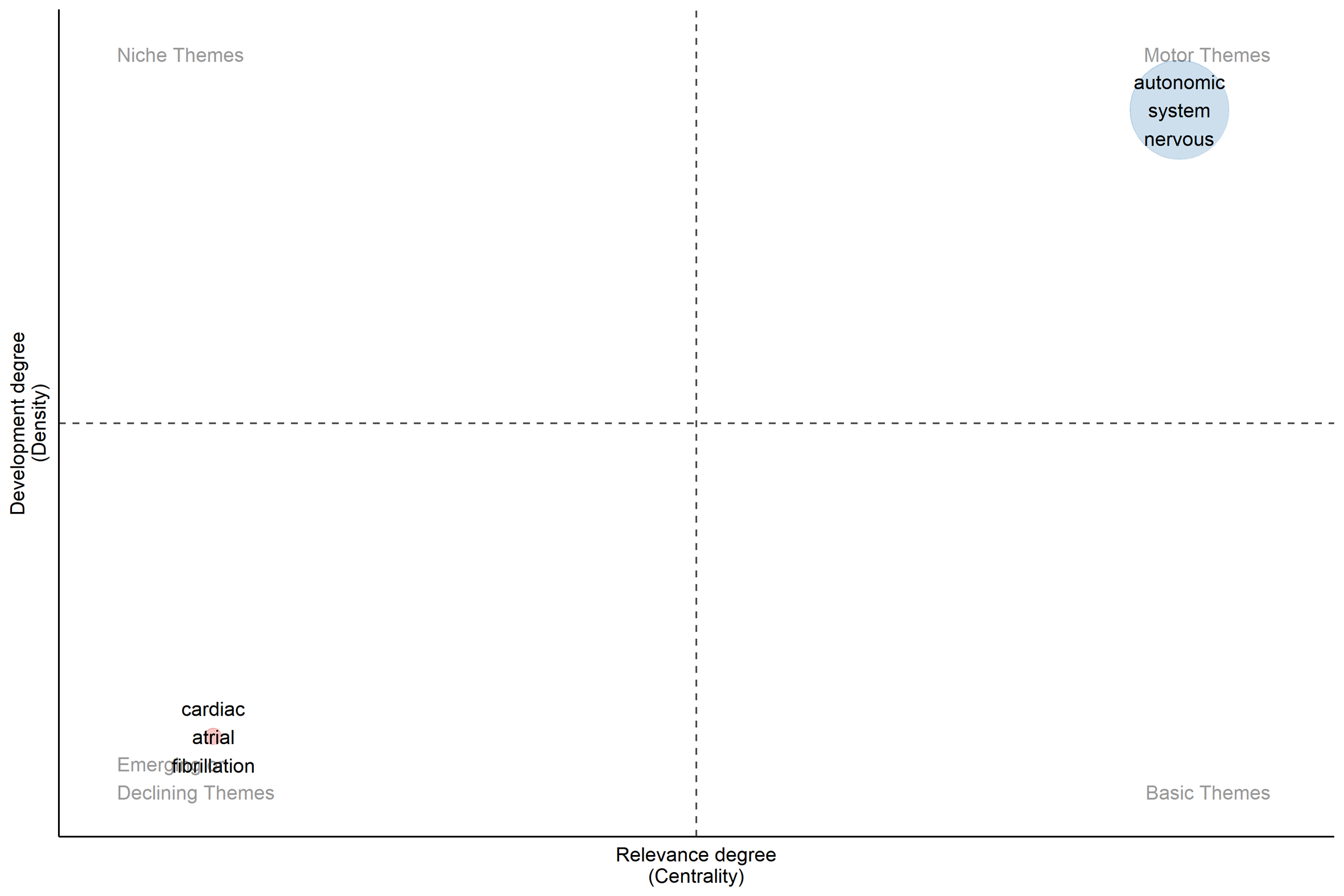

Supplement: Supplementary file 3 [file Image_3.TIF]

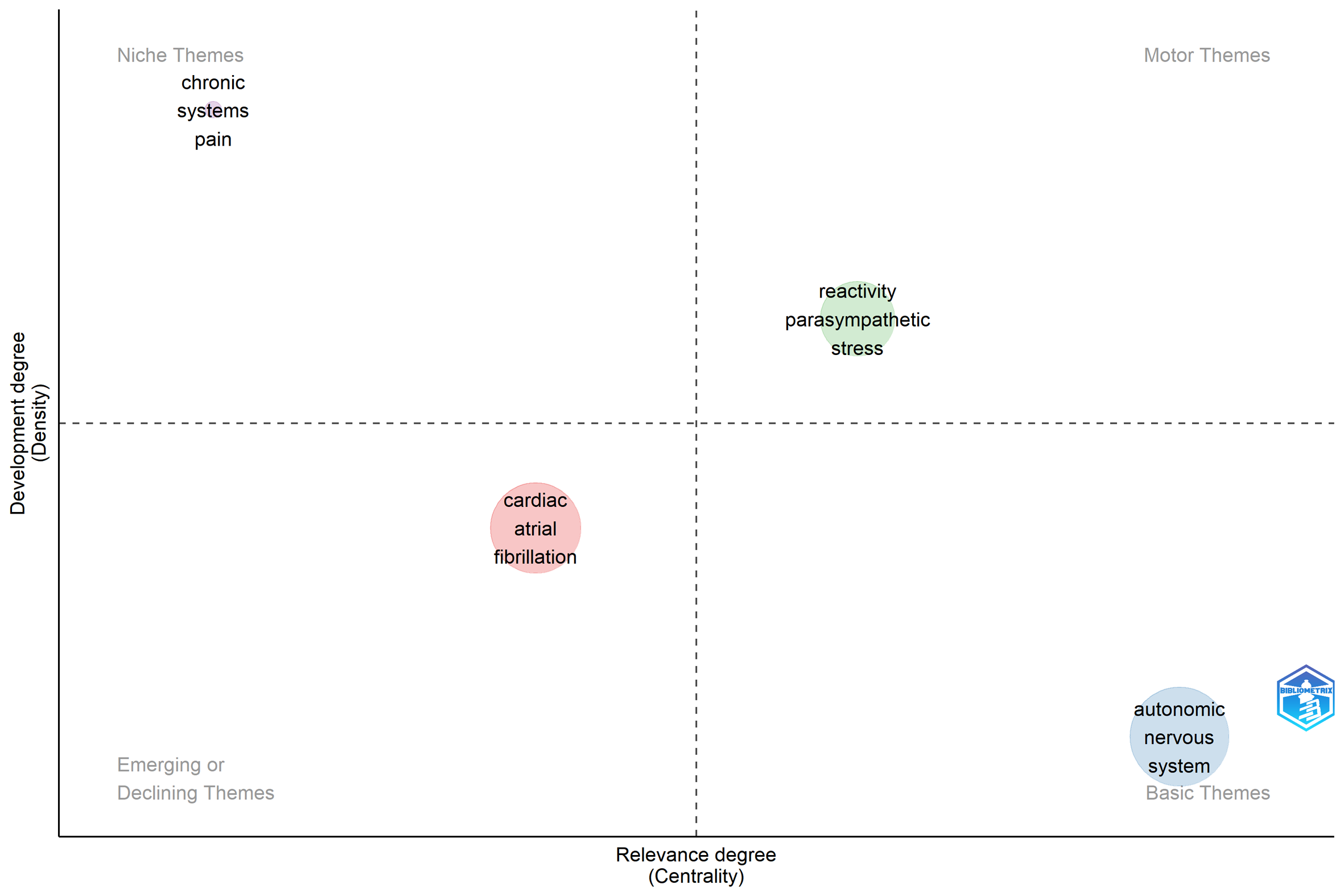

Supplement: Supplementary file 4 [file Image_4.TIF]
