# Supplementary material for: The developments and emerging trends of Autonomic Nervous System Research in Arrhythmia: a bibliometric study from 2004 to 2024
Source: Front Neurosci. 2025 Apr 28;19:1595253. doi: 10.3389/fnins.2025.1595253 (PMC12066699; doi:10.3389/fnins.2025.1595253)
Supplement: Supplementary file 5 [file Table_1.docx]

Supplementary Table 1 Inclusion Criteria

| Type | Inclusion Criteria |
| --- | --- |
| Research Content Standards | The core research themes of the literature should be closely related to the autonomic nervous system and arrhythmia, focusing on the value of autonomic nervous function changes in at least one aspect of arrhythmia, such as its etiology, diagnosis, treatment, or prognosis assessment. |
| Literature Quality Standards | The literature must contain at least five pages; reports or short papers with fewer than five pages will not be included. |
|  | The literature must have complete information elements (including abstract, author information, keywords, references, etc.). If key information elements are incomplete, the paper will not be included. |
|  | The literature must undergo strict and standardized single-blind or double-blind peer review. Studies that have not undergone single-blind or double-blind peer review will not be included. |

Supplementary Table 2 According to Bradford’s law, the 574 journals were classified into zones 1-3

| Zone | No. of journals | No. of publications | Percentage |
| --- | --- | --- | --- |
| 1 | 30 | 462 | 33.41% |
| 2 | 139 | 466 | 33.69% |
| 3 | 405 | 455 | 32.90% |
| Total | 574 | 1383 | 100%% |

Supplementary Table 3 Information about all keyword clusters

| Cluster-ID | Label | Size | Silhouette | Mean [year] |
| --- | --- | --- | --- | --- |
| 0 | Respiratory sinus arrhythmia | 134 | 0.818 | 2014 |
| 1 | Atrial fibrillation | 125 | 0.732 | 2012 |
| 2 | Heart failure | 117 | 0.719 | 2012 |
| 3 | Heart rate | 95 | 0.767 | 2007 |
| 4 | Orthostatic intolerance | 78 | 0.703 | 2014 |
| 5 | Major depressive disorder | 73 | 0.736 | 2013 |
| 6 | Forensic pathology | 9 | 0.987 | 2005 |
